# The role of hepatic myofibroblasts in liver cirrhosis in fallow deer (*Dama dama*) naturally infected with giant liver fluke (*Fascioloides magna*)

**DOI:** 10.1186/1746-6148-9-45

**Published:** 2013-03-06

**Authors:** Darko Marinković, Vladimir Kukolj, Sanja Aleksić-Kovačević, Milijan Jovanović, Milijana Knežević

**Affiliations:** 1 Department of Pathology, Faculty of Veterinary Medicine, Univeristy of Belgrade, Bulevar oslobodjenja 18, Belgrade, Serbia

**Keywords:** Deer, Liver, Liver fluke, Myofibroblasts, Immunohistochemistry

## Abstract

**Background:**

This paper describes liver cirrhosis in 35 fallow deer infected with the giant liver fluke, as well as the distribution, origin, and role of myofibroblasts in its development.

**Results:**

In liver of infected deer, stripes of connective tissue are wound around groups of degenerated and regenerated liver lobuli. In the connective tissue, lymphocytes and macrophages which often contain parasite hematin are also present. The walls of the bile ducts are thickened, the epithelium multiplied with mucous metaplasia, and desquamated cells, parasite eggs and brown pigment are present in their lumen.

In the livers with cirrhosis, immunopositivity to α-SMA and desmin was observed in cells in portal and septal spaces, at the edge between fibrotic septa and the surrounding parenchyma and in perisinusoidal spaces. These cells vary in size, they are round, oval, spindle-shaped or irregular in shape, similar to vascular smooth muscle cells. The derangement of epithelial-mesenchymal interactions detected in chronic cholangiopathies is most probably the pro-fibrogenic mechanism in liver cirrhosis of fallow deer (*Dama dama*) infected with the giant liver fluke (*Fascioloides magna*).

**Conclusion:**

Myofibroblasts, especially hepatic stellate cells (HSCs), play an important role in the synthesis of extracellular matrix components in the development of parasitic fibrosis and cirrhosis in the liver of fallow deer.

## Background

The giant liver fluke (*Fascioloides magna*) is a parasite which, contrary to other liver flukes of ruminants, exists as parasite in the hepatic parenchyma of domestic and wild ruminants, where it inflicts damage to all liver structures with the consequent development of liver fibrosis and cirrhosis. The giant liver fluke originates from North America, from where it spread to other regions through imports of deer game. It occurs sporadically in Central Europe, and its spreading to Serbia is probably connected with the course of the Danube River, as its flooding spreads the intermediate host, the dwarf pond snail *Galba truncatula*[1-5].

Through its penetration and spreading through the liver in the early stage of the disease, the giant liver fluke causes changes in the liver parenchyma that are characterized by findings of cystic spaces. These cystoid formations are filled with brown mucous fluid which contains parasites of oval leaf-like shape, around 50–70 × 30-40 mm in diameter and dark red in colour, without a cephalic cone, and with a clearly expressed mouth sucker. The thin-walled cystoid formations partly communicate with the bile pathways through which the liver fluke releases eggs which are disseminated into the outer environment by faeces. In addition to the presence of the liver fluke, there is also visible pigmentation in the form of black spots in the liver parenchyma, the portal lymph node, the peritoneum, diaphragm, and omentum [1]. The microscopic finding in the liver of infected deer includes the presence of large quantities of connective tissue that forms wider or narrower stripes that are wound around groups of degenerated and regenerated liver lobuli which are irregular in shape and of unequal size. Connective tissue septa are often seen to contain lymphocytes, macrophages which often contain brown pigment – parasite hematin, which is also present in the parenchyma. The bile duct walls are thickened, and desquamated cells, parasite eggs and brown pigment are present in their lumen [1,6-9].

These changes are somewhat similar to those described in the liver of domestic ruminants in connection with infection with the big liver fluke *Fasciola hepatica*. In chronic form, changes in infections with *F. hepatica* include stenosis of portal blood vessels, hyperplasia of blood vessels *tunica media* and a large number of newly-created blood vessels in the connective tissue stripes. Liver fibrosis and cirrhosis develop as the ultimate result of chronic liver damage caused by *F. hepatica *[10-13].

As it is known, hepatic fibrosis and cirrhosis develop following chronic damage to parenchymatous cells caused by infective agents, toxins, drugs, chemicals, malnutrition, metabolites, and hypoxia [14-17]. During the process of fibrosis and cirrhosis development, the loss of hepatocytes leads to fibroblast proliferation and transdifferentiation [18-27]. Accordingly, myofibroblast-dependent progressive fibrogenesis is sustained by at least three main pro-fibrogenic mechanisms: 1) chronic activation of the wound healing response, 2) radical oxygen system and other oxidative stress-related reactive mediators, 3) dearangement of epithelial-mesenchymal interaction and epithelial-mesenchymal transition detected in chronic cholangiopathies [28,29]. Based on location and immunohistochemical profile three myofibroblasts (MF) subpopulations were described. These comprise 1) portal or septal MFs, present in the portal areas or in newly formed fibrous septa, 2) interface MFs, present at the interface between parenchyma and stroma of the portal areas or newly formed fibrous septa, and 3) the perisinusoidally located hepatic stellate cells (HSCs) [22,28]. All types have fibrogenic potential, but many investigators regard HSCs as the principal fibrocompetent cell in the liver. Depending on the primary site of injury the resulting fibrosis may be restricted to the portal areas, as in most biliary diseases, or may be present in the hepatic parenchyma as seen in chronic hepatitis and cirrhosis. Although incompletely understood, the activation or transdifferentiation of MFs is a key event in liver tissue repair. Activated HSC express α-SMA and desmin and in humans and rats are without doubt the most important cells that are involved in the creation of extracellular matrix which occurs within liver fibrosis [30-40].

The behaviour of hepatic MFs during the process of development of parasitic liver cirrhosis in connection with natural infection of fallow deer (*Dama dama*) with the giant liver fluke (*Fascioloides magna*) is not known. This fact was the reason why the distribution and localization of hepatic MFs have been described and their role discussed in this paper, in addition to the descriptions of histological characteristics of parasitic liver cirrhosis in fallow deer.

## Material and methods

### Animals

Livers of 67 fallow deer of different sex and age have been analyzed in this paper. All the samples in this research were collected from animals that had been shot as part of a program to control the deer population. The material originated from deer shot within sanitary regulations and under veterinarian and biologist supervision in area of Northern Serbia closed to river Danube. The criteria for the selected deer were: poor condition of the animal, sluggish movement, poorly developed musculature, low representation of fatty tissue, poor hair cover and diarrhoea.

### Histopathology and immunohistochemistry analysis

Liver samples were fixed in 10% buffered formalin, and, after standard processing in an automated tissue processor, cast in paraffin blocks. Paraffin sections 3–5 μm thick were stained with hematoxylin and eosin and with Masson’s trichrome for connective tissue for light microscopic examination.

Primary antibodies α-SMA and desmin were chosen for the study of expression of activated HSC in three-step indirect immunohistochemical technique. After antigen retrieval and inactivation of endogenous peroxidase, sections were incubated with appropriate primary antibodies diluted in PBS. Primary antibodies used for immunohistochemistry were Desmin D33 (DAKO, M0760) diluted 1/100 and α-SMA 1A4 (DAKO, M0851) diluted 1/50. All rinsing procedures and serum dilutions were done in PBS (pH 7.2). The detection kit was labeled streptavidin biotin (LSAB2) System-HRP, Rabbit/mouse (DAKO, K0675). Reactions were visualized by using 3,3-Diaminobenzidine (DAB+) (DAKO, K3468) and counterstaining with hematoxylin. Smooth muscle cells within the blood vessel wall were used as internal positive controls for α-SMA and desmin.

Liver sections not treated with the primary antibody were used as negative controls.

## Results

### Macroscopic findings

**Figure 1:**
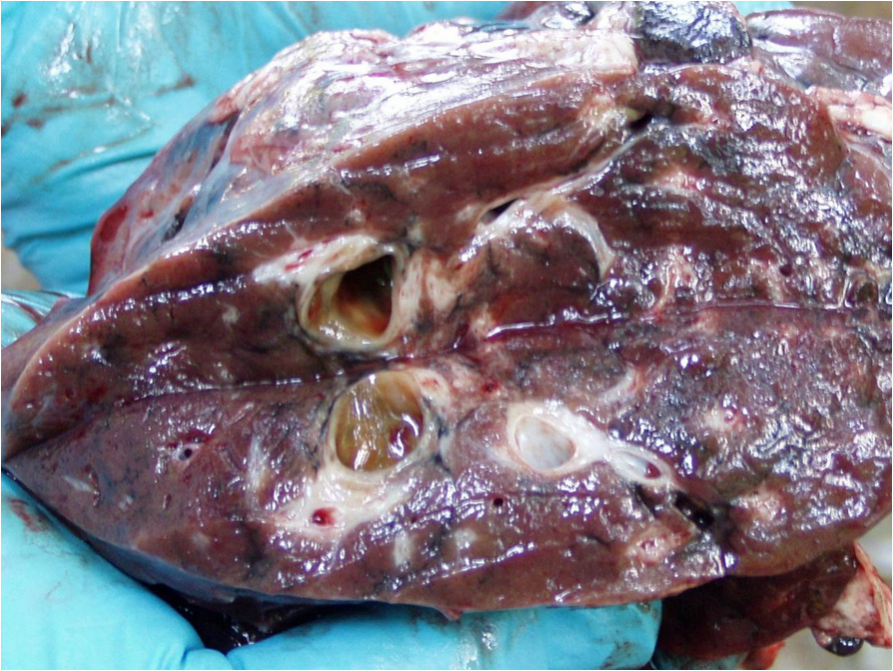
Section of the deer liver, dirty-grey in colour, with accumulated fibrin on the surface and thick-wall cystoid formations on the cross-section and circumscribed deposits of black pigment.

In 35 (52.24%) animals, macroscopic changes were observed in the liver. The livers were enlarged, dirty-grey in colour partialy with black pigment deposits, and with accumulated fibrin on the surface and cystoid formations on the cross-section. Texture was mostly rubbery and tough, except in places where cystoid expansions were observed (Figure 1). These cystoid formations were filled with a brown mucous liquid which contained parasites of oval, leaf-like shape, around 50-70 mm × 30-40 mm in diameter, dark red in colour, without a cephalic cone, and with a clearly expressed cephalic sucker. The identification of the giant liver fluke (*Fascioloides magna*) was confirmed also by parasitological analysis.

### Histopathology

**Figure 2:**
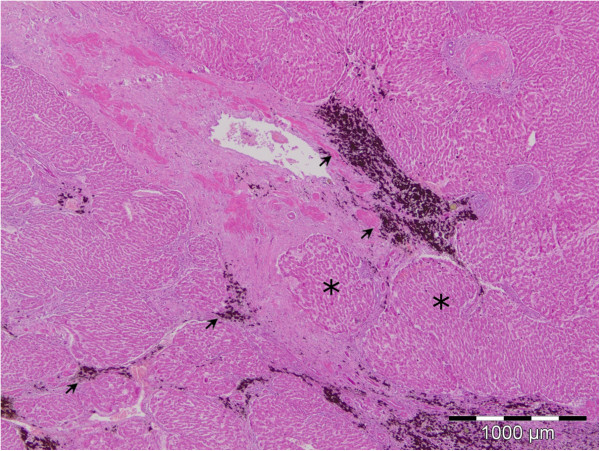
**Disrupted architecture of the liver with cirrhosis.** Connective tissue fibres present in the form of wide or narrow stripes divide the liver parenchyma into pseudolobuli of unequal size and irregural shape (asterisk). Deposits of hematin pigment (arrow), HE.

The histopathological examination of the deer liver showed that the liver architecture was partly or completely disrupted with numerous pseudolobuli (Figure 2). Connective tissue fibres were present in the form of wide or narrow stripes and they divide the liver parenchyma into pseudolobuli of unequal size and irregular shape. In the multiplied connective tissue stripes, there were numerous blood vessels with thickened walls which contain smooth muscle cells, partly vacuolized, and partly with homogenized cytoplasm.

**Figure 3:**
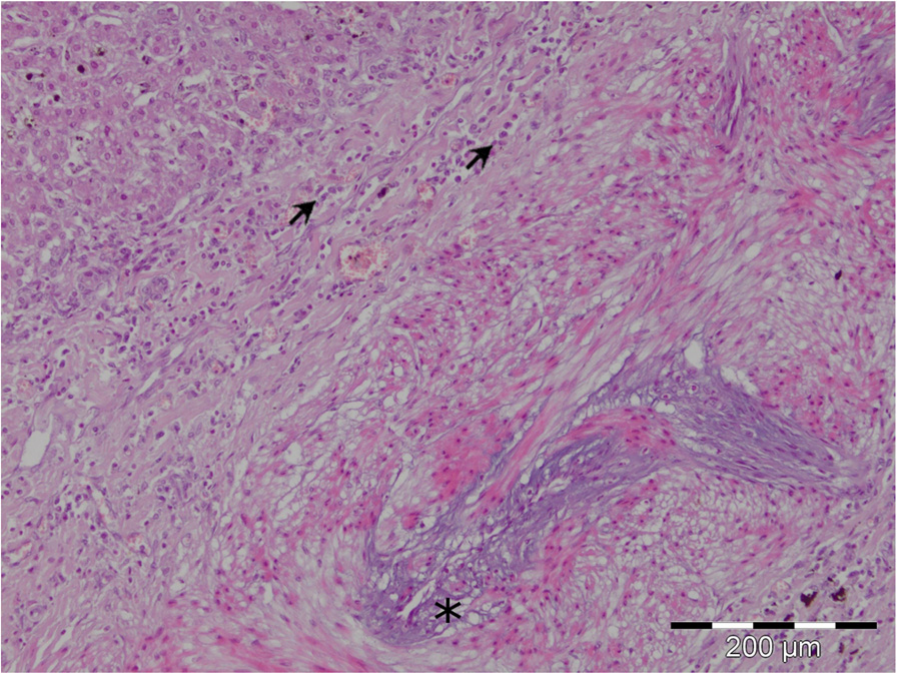
**Vacuolized endothelial cells of the blood vessel (asterisk).** Cellular infiltrate in the vessel wall comprised of macrophages and eosinophil granulocytes (arrow), HE.

Endothelial cells of the blood vessels were also vacuolized, and sometimes necrotized and they were found free in the lumen. In close proximity to the altered blood vessels there was cellular infiltrate comprised of macrophages and eosinophil granulocytes. Groups or individual smooth muscle cells were sometimes observed in the connective tissue stripes, extravascularly (Figure 3).

The connective tissue stripes contained numerous bile ducts of different diameter and individual epithelial cells similar to the epithelium of bile ducts. Bile duct epithelium-like epithelial cells were also seen between hepatocytes. Regenerated hepatocytes were organized into pseudolobuli of different size and of irregular, most often oval shape, without a central vein. The presence of macrophages filled with brown pigment was observed between hepatocytes and in the connective tissue stripes, and the brown pigment was sometimes also seen free in the connective tissue. The wall of certain bile ducts was thickened, and the epithelium proliferated to the form of edematous swelling and mucous metaplasia with a large number of cup-shaped cells. In addition to the described changes, a number of the samples were also seen to contain well encapsulated parasite cystoid formations in the liver parenchyma. These cystoid formations were characterised by a thick connective tissue capsule composed of fibroblasts, connective tissue fibers between which macrophage, lymphocytes, eosinophil granulocytes, and a smaller or larger number of cells with brown pigment were situated. Within this connective tissue capsule, there was necrotic detritus, fibrin, brown pigment, and remains of parasites. Close to the cystoid formations, hepatocytes were atrophic, and the liver lobes and pseudolobuli deformed.

### Immunohistochemistry

In the sections of control deer livers, cells positive to α-SMA and desmin antibodies were scattered, diffusely, through the entire liver parenchyma. The positive reaction of pericytes and smooth muscle cells of terminal and sublobular venous blood vessels were observed. In the portal triads, a positive reaction was observed in the arterial *tunica media*, and slighter positivity was in the walls of the portal veins. Rare immunopositive cells were observed in Glisson’s capsule.

**Figure 4:**
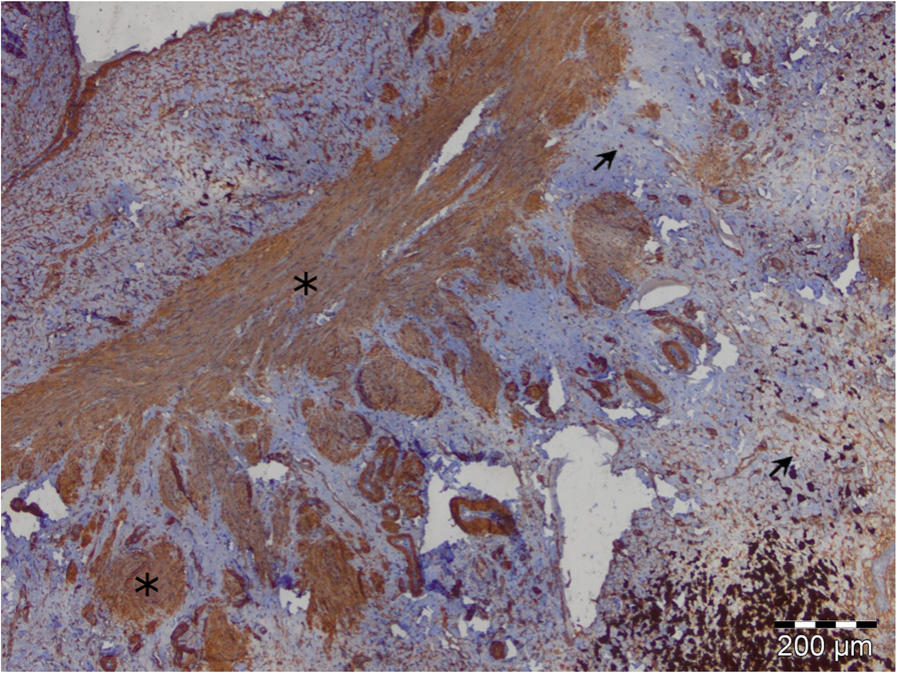
Perisinusoidal star-like shape α-SMA positive HSCs with homogenous cytoplasm and long cytoplasmatic extensions (arrow) and elongated α-SMA positive MFs in fibrotic septa (asterisk), LSAB2.

**Figure 5:**
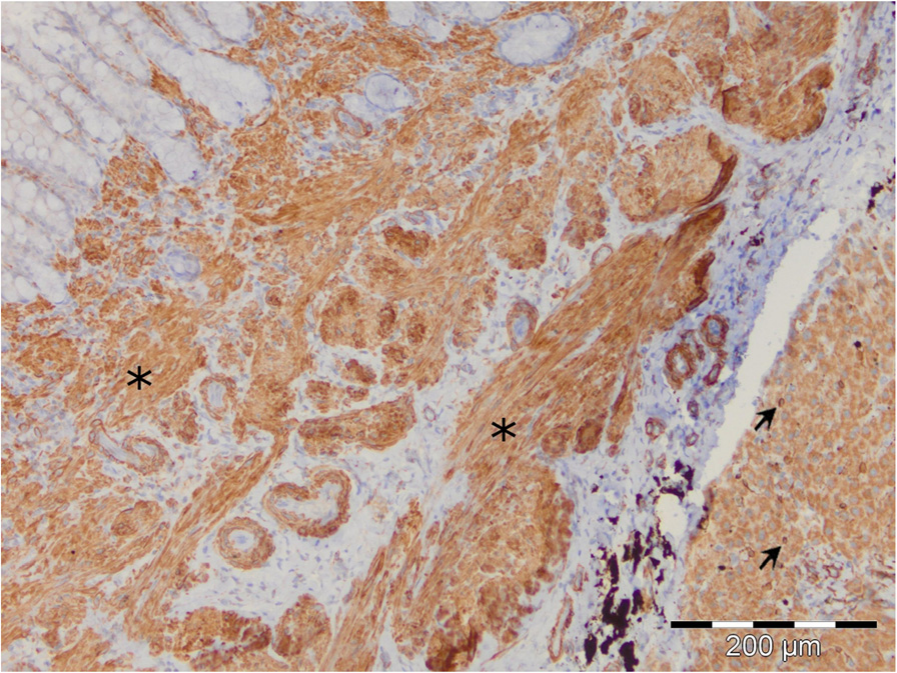
Perisinusoidal star-like shape desmin positive HSCs in liver parenchyma (arrow) and elongated desmin positive MFs in fibrotic septa (asterisk), LSAB2.

In the livers with cirrhosis, immunopositivity to α-SMA (Figure 4) and desmin (Figure 5) was observed on cells in portal and septal spaces, at the edge beetween fibrotic septa and the surrounding parenchyma and in perisinusoidal spaces. Immunopositivity to α-SMA and desmin was observed also on the walls of blood vessels of the portal space, as well as in the increased septal connective tissue. Vascular smooth muscle cells and individual cells situated in connective tissue stripes were also strongly positive to α-SMA and desmin in livers with cirrhosis.

Perisinusoidal α-SMA and desmin positive cells were recognized by their star-like shape, homogenous cytoplasm without vacuolization and long cytoplasmatic extensions which exibit a fine granular reaction. Morphological polymorphism was most expressed in α-SMA and desmin positive cells that are located in connective tissue stripes of livers with cirrhosis. These cells were round, oval, spindle-shaped, or irregular in shape, of varying sizes. Most of these cells, according to their morphological and phenotype characteristics, are similar to vascular smooth muscle cells. Elongated immunopositive cells with a poorly developed body and short cytoplasmatic extensions were found on the boundary between fibrotic septa and the surrounding parenchyma.

The absence of cells positive to both examined markers was observed on the periphery of regenerative lobuli that occur in liver cirrhosis.

Endothelial cells of liver blood vessels, hepatocytes and bile duct epithelium showed a negative reaction to the examined markers.

## Discussion

It was proved that the giant liver fluke is present in the fallow deer population in the territory of Serbia. The finding of this parasite in the fallow deer population represents a threat as the giant liver fluke could spread to domestic ruminants which reside in close proximity to hunting grounds [1,4,5,7-9].

This paper is focused on pathohistological changes in the liver of fallow deer with parasitic cirrhosis caused by the giant liver fluke and the heterogeneity of the hepatic myofibroblast population.

Changes corresponding to liver cirrhosis were established in 35 (52,24%) of the 67 examined fallow deer livers, with alterations in all liver structures, including the parenchyma, vascular and biliary system. The accumulation of extracellular matrix in the liver in the form of narrow or wide stripes, the forming of pseudolobuli and the presence of chronic cellular infiltrate consisting of lymphocytes and macrophages is accompanied by the presence of macrophages filled with parasite hematin and is a consequence of the host blood metabolism process in the parasite. Furthermore, the determined cystoid formations localized in the parenchyma and the morphological characteristics of the parasite are confirmation that these are changes caused by the giant liver fluke *F. magna*, and not by *F. hepatica* or *Dicrocoelium dendriticum* which primarily damage the intrahepatic biliary system [1,5,12,13]. Concerning the fact that cysts have only occasionally an epithelium covering part of the inner surface the term cystoid formation is more appropriate [5]. Livers of several fallow deer were found to contain proliferation of the epithelium of bile ducts which are framed with a large quantity of connective tissue. Hyperplasia of the bile duct epithelium is described in literature within infection with *F. hepatica *[12]. It is also possible to link the analogous changes found in fallow deer with proline synthesis and secretion on behalf of the parasite *F. magna* as it occurs in infection with *F. magna *[11]. Vascular changes are a consequence of traumatic phlebitis and increased accumulation of connective tissue. The compensatory increased arterial blood inflow leads to hyperplasia and hypertrophy of the *tunica media *[12,13].

It is possible to explain the increased amount of extracellular matrix, occurring as a consequence of the accumulation of different proteins, during myofibroblast activation, migration and accumulation.

Imunohistochemical staining of cirrhotic livers proved that α-SMA and desmin positive cells present a heterogenic cell population regarding morphology and distribution. Progressive fibrillogenesis in fallow deer livers with parasite cirrhosis is most probably a consequence of the stepped up expression of α-SMA and desmin on portal, septal, interface, and perisinusoidal MFs. The established strong immunopositivity in myofibroblasts of connective tissue stripes, hyperplastic *tunica media*, as well as in extravascular smooth muscle cells is viewed by certain authors as a consequence of their role in pathological angiogenesis during the progression of chronic liver damage [28]. Our study has shown that hepatic MFs in fallow deer livers are a very heterogenic cell population, both regarding distribution and regarding morphology, similar as in humans and other animal species [18-27]. It is known that, in addition to MFs, other cell populations in the liver, such as damaged hepatocytes, activated Kupffer cells, and endothelial cells of sinusoids, also play a significant part in fibrogenesis [28].

The derangement of epithelial-mesenchymal interactions detected in chronic cholangiopathies is most likely the pro-fibrogenic mechanism in liver cirrhosis in fallow deer (*Dama dama*) infected with the giant liver fluke (*Fascioloides magna*). This is the first publication reporting morphological changes in liver in fallow deer (*Dama dama*) in Serbia.

## Conclusion

Myofibroblasts, especially HSCs, play an important role in the synthesis of extracellular matrix components in the development of parasitic fibrosis and cirrhosis in the liver of fallow deer. Activated HSCs, as well as portal and septal myofibroblasts, correlate with the degree of liver fibrosis.

## Abbreviations

α-SMA: Alpha-smooth muscle actin; MFs: Myofibroblasts; HSCs: Hepatic stellate cells

## Competing interests

Hierby we disclose any financial and personal relationships with other people or organisations that could inappropriately influence our work.

## Authors’ contributions

VK and MK contributed to conception and design, data analysis, drafting and writing of the manuscript. DM and MJ contributed to necropsy and collection of samples and histopathological analysis. SAK and VK contributed to pathological, histopahtological and immunohistochemical analyses. All authors have read and approved the final manuscript.

## Authors’ information

Department of Veterinary Pathology, Faculty of Veterinary Medicine, University of Belgrade, Belgrade, Serbia, Bulevar oslobodjenja 18, str. 11000 Belgrade, Serbia.
